# DmdA‐independent lag phase shortening in *Phaeobacter inhibens* bacteria under stress conditions

**DOI:** 10.1111/febs.70128

**Published:** 2025-05-03

**Authors:** Delia A. Narváez‐Barragán, Martin Sperfeld, Einat Segev

**Affiliations:** ^1^ Department of Plant and Environmental Sciences Weizmann Institute of Science Rehovot Israel; ^2^ Present address: Institute of Microbiology, ETH Zurich Switzerland

**Keywords:** dimethylsulfoniopropionate, functional redundancy, Lag phase shortening, methionine synthase, osmotic and oxidative stress, *Phaeobacter inhibens*

## Abstract

Bacteria can shorten their lag phase by using methyl groups from compounds like dimethylsulfoniopropionate (DMSP), which are incorporated into cellular components via the methionine cycle. However, the role of specific methionine synthases in this process is not fully understood. Using transcriptomics, genetics, and biochemical assays, we investigated methionine synthases involved in lag phase shortening in *Phaeobacter inhibens*. We focused on a cobalamin‐dependent methionine synthase (MetH)‐like complex encoded by three genes: a betaine‐homocysteine S‐methyltransferase (*bmt*), a cobalamin‐binding protein (*cbp*), and an intermediate methyl carrier (*PGA1_c16040*). Expression profiling revealed transcriptional decoupling among these genes. Deleting *bmt* disrupted lag phase shortening in response to DMSP. Functional assays showed that Bmt can directly produce methionine from DMSP and betaine, independent of tetrahydrofolate (THF) or cobalamin. Interestingly, under stress conditions, lag phase shortening occurred even in the absence of dimethylsulfoniopropionate demethylase DmdA, the primary DMSP demethylase. Under osmotic and oxidative stress, *bmt* expression increased significantly in response to both DMSP and betaine, suggesting an alternative methylation route. This highlights the role of Bmt as both demethylase and a methionine synthase under stress, offering a cost‐effective strategy for methyl group assimilation. Our findings reveal a novel stress‐responsive pathway for methionine synthesis and demonstrate the role of Bmt in promoting bacterial adaptation by accelerating the lag phase.

## Graphical abstract

The lag phase is the preparatory period during which bacteria transition from dormancy to active cell division, and is thus crucial for resuming bacterial growth. It can be shortened by fueling the methionine cycle with methyl groups from dimethylsulfoniopropionate (DMSP) or other methylated compounds. While DmdA is the primary DMSP demethylase in *Phaeobacter inhibens*, stress activates additional enzymes like Bmt. This enzymatic flexibility allows bacteria to exit the lag phase rapidly, highlighting the importance of lag phase shortening and enzyme flexibility in adaptation to environmental changes. Our findings reveal a novel stress‐responsive pathway for methionine synthesis in promoting bacterial adaptation.

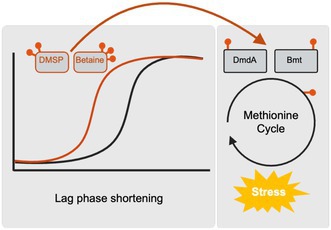

**Abbreviations**
B_12_
: cobalamin
Bmt: betaine methyltransferase
cbp: cobalamin‐binding protein
CH_3_‐B_12_
: methylcobalamin
CH_3_‐THF: methyltetrahydrofolate
core‐MetE: minimal versions of MetE
dmdA: dimethylsulfoniopropionate‐dependent demethylase
DMSP: dimethylsulfoniopropionate
dNTPs: deoxynucleotide triphosphates
H_2_O_2_
: hydrogen peroxide
MetE: cobalamin‐independent methionine synthase
MetH: cobalamin‐dependent methionine synthase
NaCl: sodium chloride
qRT‐PCR: real‐time quantitative reverse transcription PCR
SAH: *S*‐adenosylhomocysteine
SAM: S‐adenosylmethionine
SMM: *S*‐methylmethionine
THF: tetrahydrofolate
TMP: trimethoprim

## Introduction

The lag phase is the preparatory period during which bacteria transition from dormancy to active cell division [1]. This crucial adaptation period is influenced by multiple factors, including inoculum size, cell history, and environmental conditions [2]. Bacteria are under constant evolutionary pressure to optimize their lag phase [3], which can be achieved by using substrates from the environment to overcome biosynthetic bottlenecks [4, 5]. Short lag phases allow bacteria to rapidly respond to environmental changes or outcompete others in dynamic ecosystems [1]. Bet‐hedging strategies, where subsets of cells quickly divide while others remain dormant, are advantageous for bacterial populations [6]. Additionally, prolonged lag phases appear to act as a defense mechanism, conferring stress tolerance, including to antibiotics [7]. The methionine cycle is a central metabolic pathway involved in regulating the length of the lag phase [4]. Bacteria can accelerate their lag phase by utilizing methyl groups from external methylated donor compounds, such as dimethylsulfoniopropionate (DMSP) and glycine betaine (hereafter referred to as betaine), and incorporate them into building blocks via the methionine cycle [4]. This cyclic pathway is responsible for the synthesis of the amino acid methionine [8], which is also essential for protein translation initiation [9] and various biosynthetic pathways [10]. Methionine synthesis is widely distributed across all domains of life, underscoring its importance in cellular metabolism, while the involved enzymes and regulatory elements exhibit evolutionary plasticity [11].

**Fig. 1:**
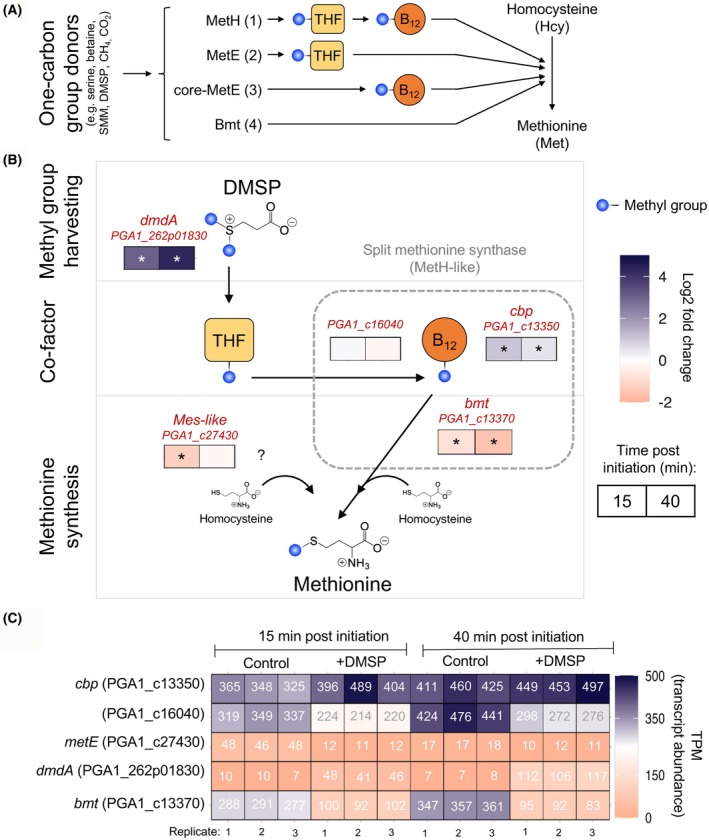
Transcriptional response of demethylation and methionine synthase genes in *Phaeobacter inhibens* bacteria elicited by DMSP during the lag phase. (A) Schematic overview of methionine synthesis reactions and the co‐factors involved: (1) MetH sequentially transfers a methyl group from methyltetrahydrofolate (CH_3_‐THF) via cobalamin (B_12_) to homocysteine. (2) MetE transfer a methyl group from CH_3_‐THF to homocysteine. (3) core‐Met transfers a methyl group from methylcobalamin (CH_3_‐B_12_) to homocysteine. (4) Direct transfer of a methyl group to homocysteine without co‐factors involved. (B) The transcriptional response of genes putatively involved in *P. inhibens* methionine synthesis was analyzed in freshly initiated bacterial cultures during the lag phase. Glucose‐grown stationary phase bacteria were transferred to fresh medium containing 1 mm glucose and supplemented with 50 μm DMSP and were compared to control cultures without DMSP. Changes in gene expression (log_2_ fold change) are indicated as colored boxes that correspond to the time post initiation—15 min (left box) and 40 min (right box). Colors show upregulation (purple) and downregulation (orange) in response to DMSP. The gene involved in DMSP demethylation (*dmdA*) was upregulated by 3.04‐ and 4.34‐Log_2_ fold at 15 and 40 min post initiation, respectively, in response to DMSP. The three genes encoding the split methionine synthase exhibited a combination of upregulation (*cbp*), no regulation (*PGA1_c16040*) and downregulation (*bmt*). The core methionine synthase‐like (*Mes‐like*) was downregulated 15 min post initiation. Asterisks indicate significant differential gene expression (adjusted *P*‐value < 0.05; log_2_ fold change < −0.585 and > +0.585). (C) Transcript abundance of methionine synthesis‐related genes during the lag phase of *P. inhibens* bacteria supplemented with 50 μm DMSP compared to control cultures. Transcript abundances are presented as log_2_ TPM (transcripts per kilobase million) of normalized paired‐end read counts. The underlying dataset was previously described in Sperfeld & Narváez‐Barragán *et al*. [4]. *B*_*12*_, cobalamin; *bmt*, betaine methyltransferase; *cbp*, cobalamin‐binding protein; *CH*_*3*_ *‐B*_*12*_, methylcobalamin; *CH*_*3*_ *‐THF*, methyltetrahydrofolate; *core‐MetE*, minimal versions of MetE; *dmdA*, dimethylsulfoniopropionate‐dependent demethylase; DMSP, dimethylsulfoniopropionate; *Hcy*, homocysteine; *Mes‐like*, core methionine synthase; *Met*, methionine; *MetE*, cobalamin‐independent methionine synthase; *MetH*, cobalamin‐dependent methionine synthase; *PGA1_c16040*, methyltetrahydrofolate—cobalamin methyltransferase; *THF*, tetrahydrofolate. Blue dots represent the transfer of the methyl group from donor DMSP to homocysteine, resulting in methionine.

Methionine synthases play a crucial role in the methionine cycle, catalyzing the methylation of homocysteine to methionine [8]. Methionine synthases can be grouped based on their co‐factor requirements (Fig. 1A). The cobalamin‐dependent methionine synthase—MetH [12]—is a large multi‐domain complex that catalyzes the sequential transfer of a methyl group from methyltetrahydrofolate (CH_3_‐THF) via cobalamin (B_12_) to homocysteine (Fig. 1A, reaction 1). In contrast, the cobalamin‐independent methionine synthase—MetE—can directly transfer a methyl group from CH_3_‐THF to homocysteine (Fig. 1A, reaction 2), but it functions with reduced rates [13]. Minimal versions of MetE were also described, named core‐MetE, which lack the tetrahydrofolate‐binding domain [14, 15] and often utilize methylcobalamin (CH_3_‐B_12_) as a methyl group donor (Fig. 1A, reaction 3) [15]. Finally, *N*‐ and *S*‐methylated substrates such as betaine [16] or *S*‐methylmethionine (SMM) [17] can serve as direct methyl group donors for homocysteine methylation, without involving intermediate methyl group‐carrying co‐factors (Fig. 1A, reaction 4). The latter methionine synthesis route is catalyzed by minimal versions of MetH, lacking the cobalamin and tetrahydrofolate‐binding domains. These simplified versions are designated Bmt (betaine methyltransferase) in other bacteria [16] and in the current study. The diversity of methionine synthases, which occurs across all domains of life, highlights the evolutionary adaptations and the importance of these enzymes in the methionine cycle [11]. The role of specific bacterial methionine synthases in the process of lag phase reduction remains unclear.

Here, we explore the involvement of methionine synthases in lag phase reduction of the model marine bacterium *Phaeobacter inhibens*, a member of the *Roseobacter* group [18, 19]. *P. inhibens* commonly interacts with microalgae [4, 5, 20, 21, 22, 23, 24, 25] is abundant in marine environments [26], and demonstrates remarkable potential for secondary metabolite production [19]. This bacterium was described to possess a split methionine synthase, in which the domains of the MetH‐like methionine synthase are separated into single proteins [27]. It was suggested these enzymes together build the canonical MetH complex, which produces methionine from CH_3_‐THF (which is produced intercellularly from e.g., serine; Fig. 1A, reaction 1). Within this complex, the methyl group is transferred by a protein encoded by the gene *PGA1_c16040* to cobalamin, the intermediate methyl carrier, along with a cobalamin‐binding protein (cbp). Finally, the methyl group is transferred from cobalamin to homocysteine via Bmt, resulting in the formation of methionine [27, 28] (Fig. 1B). In ocean environments, phytoplankton‐derived *N*‐ and *S*‐methylated compounds, such as betaine and DMSP, are abundant [29, 30]. As *P. inhibens* is a marine bacterium commonly associated with phytoplankton [20, 21, 22], the presence of a metabolic shortcut is plausible. Homologs of the Bmt enzyme have been found across various organisms and are capable of catalyzing the direct methyl group transfer from different methylated compounds to homocysteine, bypassing the use of tetrahydrofolate (THF) or cobalamin. In humans, the Bmt homolog BHMT is known to facilitate methionine synthesis by transferring methyl groups from *N*‐methylated betaine or *S*‐methylated sulfobetaine (DMSA) [31, 32]. In the soil bacterium *Sinorhizobium meliloti*, Bmt shows a similar substrate range [16]. The Bmt homolog in *E. coli* (MmuM [17, 33]) and the human BHMT‐2 enzyme [34] can transfer a methyl group directly from *S*‐methylmethionine (SMM), whereas mammalian Bmt homologs utilize DMSP as a methyl group donor [35, 36]. Notably, in environmental samples, transcripts of *bhmt* (*bmt*) from the abundant marine bacteria SAR11 were detected, although genes that encode for both the MetE and the MetH enzymes were absent in these bacteria [37]. This suggests the direct utilization of methyl groups from betaine for methionine synthesis *in situ*. Moreover, this pathway also appears to be present in other marine bacteria [37]. In *P. inhibens*, DMSP‐dependent methionine synthesis involves DmdA transferring a methyl group from DMSP to THF, producing CH3‐THF, which feeds into the MetH‐like split methionine synthase complex [4]. Bmt has the potential to act as a stand‐alone enzyme, transferring methyl groups directly from DMSP or betaine to homocysteine, producing methionine without involving the co‐factors THF or cobalamin (Fig. 1A, reaction 4).

In the presence of these compounds, Bmt has the potential to act as a stand‐alone enzyme that transfers methyl groups directly from external betaine or DMSP to homocysteine, producing methionine without involving the co‐factors THF or cobalamin (Fig. 1A, reaction 4).

In this study, we employed transcriptomics, genetic manipulation, and biochemical assays to assess the transcriptional response and the involvement of the individual methionine synthase components of *P. inhibens* during DMSP‐induced lag phase shortening [4]. Our results reveal that *in vitro*, Bmt can function as a stand‐alone methionine synthase that demethylates DMSP and betaine for direct homocysteine methylation. Furthermore, *in vivo*, in the absence of the primary DMSP demethylase DmdA, our data demonstrate that other enzymes contribute to lag phase shortening under stress conditions. Under these stress scenarios, the expression of *bmt* is upregulated during the lag phase, pointing to its possible involvement in shortening the lag period in response to DMSP and betaine. Bmt might therefore represent an alternative route for the assimilation of methyl groups from DMSP and betaine, particularly under unfavorable conditions. These findings contribute to our understanding of the enzymatic mechanisms underlying the bacterial ability to shorten the lag phase under various environmental conditions and underscore the centrality and flexibility of bacterial methionine synthesis.

## Results

### Regulatory dynamics of methionine synthase genes in response to DMSP during the lag phase

We first examined the transcriptional expression patterns of genes putatively involved in methionine synthesis during the lag phase once stationary bacteria are transferred to a fresh medium. Cultures were sampled during the first 15 and 40 min of the lag phase after bacteria were transferred to fresh medium containing 1 mm glucose as the sole carbon source. Cultures were either supplemented with DMSP (50 μm) or untreated as control. As outlined in the introduction, it was previously shown that DMSP‐dependent methionine synthesis in *P. inhibens* involves a reaction where DmdA transfers a methyl group from DMSP to THF, producing CH_3_‐THF. This methylated THF then serves as a substrate for the MetH‐like split methionine synthase complex. Within this complex, the methyl group is transferred by a protein encoded by the gene *PGA1_c16040* to cobalamin, the intermediate methyl carrier, along with a cobalamin‐binding protein (cbp). Finally, the methyl group is transferred from cobalamin to homocysteine via Bmt, resulting in the formation of methionine (Fig. 1B) [27, 28]. Our RNA‐sequencing data revealed that the DMSP demethylase gene *dmdA* was upregulated by at least 3‐Log_2_ fold, while the transcription levels of the three split methionine synthase genes were variable, showing no apparent co‐regulation (Fig. 1B,C, and Tables S1 and S2). Observed expression patterns ranged from DMSP‐induced upregulation (*cbp*) to no regulation (*PGA1_c16040*) and downregulation (*bmt*). The key gene for methionine synthesis (*bmt*) was significantly downregulated in response to DMSP at both examined timepoints. At first look, *bmt* downregulation appears contradictory to the involvement of Bmt in DMSP methyl group assimilation. However, it is in accordance with reports on negative feedback regulation of methionine cycle components in response to their own products [10, 38, 39]. Additionally, cells have evolved mechanisms to sense methionine abundance, which allows for the regulation of *bmt* enzyme expression based on the cellular methionine demand. Alternatively, *bmt* expression could be downregulated if other enzymes are compensating for its activity or if the cells are exposed to specific molecules [10, 38, 39, 40, 41]. The downregulation of *bmt* could thus prevent the accumulation of inhibitory methionine, which we previously showed extends the lag phase of *P. inhibens* [4].

### The methionine synthase Bmt is involved in lag phase shortening in response to DMSP

**Fig. 2:**
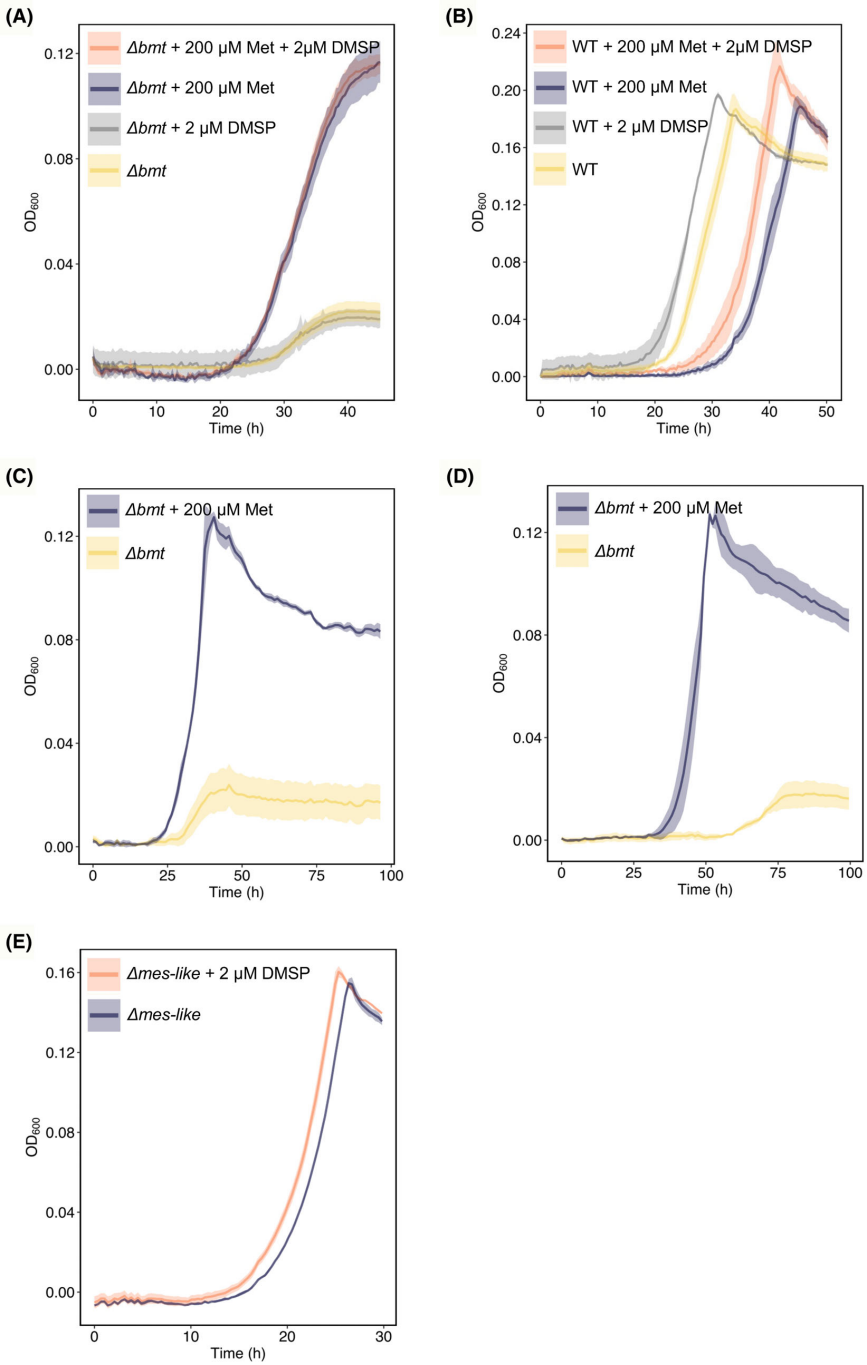
The *Phaeobacter inhibens* methionine synthase gene *bmt* is involved in lag phase shortening. (A) Growth of the ∆*bmt* mutant on 1 mm glucose was impaired in the absence of methionine (yellow) but was restored upon adding 200 μm methionine (Met) to the medium (purple). The supplementation of bacteria with 2 μm DMSP did not affect the lag time of the ∆*bmt* mutant, neither in the presence of methionine (orange), nor in its absence (gray). For statistical significance, see Table S3. (B) Lag phase of the WT strain was delayed in the presence of 200 μm methionine (purple) compared to the control without methionine supplementation (yellow). The addition of 2 μm DMSP induced lag phase shortening in both the control (gray) and methionine‐supplemented (orange) conditions. For statistical significance see Table S4. (C) The ∆*bmt* mutant exhibits low but sustained growth without methionine supplementation. Growth was monitored for 96 h with (purple) and without (yellow) 200 μm methionine. (D) Cells from (C) where highly diluted (OD_600_ 0.000001) into fresh media and continue to exhibit growth. (E) The *P. inhibens* methionine synthase Mes‐like, is not involved in lag phase shortening. The *∆mes‐like* mutant still exhibited lag phase shortening upon supplementation with 2 μm DMSP (orange), compared to control cultures (purple). *WT*: wild‐type, Δ*bmt*: betaine methyltransferase mutant, Δ*mes‐like*: core methionine synthase mutant. Lines represent the average growth curve based on three biological replicates and shaded areas indicate the standard deviation (SD).

To further investigate the mechanism by which DMSP methyl groups are utilized for methionine synthesis, we wished to evaluate whether Bmt plays a role in DMSP‐driven lag phase shortening in *P. inhibens* bacteria. Therefore, we deleted the *bmt* gene and analyzed bacterial growth and lag phase shortening capabilities. The *Δbmt* mutant is an auxotroph for methionine and therefore growth experiments were initiated with bacteria that were pre‐cultivated with 1 mm glucose and 200 μm methionine until reaching stationary phase. Indeed, methionine was required to restore growth of the *Δbmt* mutant (Fig. 2A). Under both conditions, with and without methionine, DMSP did not induce shorter lag times in the *Δbmt* mutant (Fig. 2A and Table S3). To rule out the possibility that the methionine addition itself prevented lag phase shortening, a similar experiment was conducted with wild‐type (WT) bacteria. Cultures of WT bacteria still exhibited lag phase shortening in response to DMSP even when supplemented with methionine (Fig. 2B and Table S4). These data suggest that while DmdA is the main DMSP demethylase, Bmt is involved in the lag phase shortening response towards DMSP.

Of note, residual growth was observed in the *Δbmt* mutant in the absence of methionine (Fig. 2A). To confirm that this was indeed growth, we first monitored the *Δbmt* mutant without methionine supplementation over an extended period (Fig. 2C). Second, the cultures that exhibited the residual growth were diluted into fresh media without methionine supplementation (Fig. 2D). Under both conditions, we observed low but measurable growth, which may be attributed to a putative second methionine synthase. Homology and structural analyses suggest that PGA1_c27430 encodes a protein that is similar to the catalytic domain of the MetE methionine synthase (Table S5, AlphaFold average model confidence of 95.44 for the MetE protein from *Roseobacter denitrificans* ATCC 33942 [42]). However, the absence of the THF‐binding N‐terminal domain suggests that it could function as a core methionine synthase (Mes‐like) and utilize alternative methyl donors, as observed in other bacteria [15]. Alternatively, unidentified methionine synthases may compensate for the absence of Bmt [43]. Therefore, to evaluate whether Mes‐like is involved in lag phase shortening, we generated a *Δmes‐like* mutant. The mutant exhibited 1.9 h (±0.03, *P*‐value 0.03) lag phase shortening upon supplementation with 2 μm DMSP (Fig. 2E). Thus, the methionine synthase Bmt, but not Mes‐like, appears to be involved in lag phase shortening in *P. inhibens* bacteria.

### Bmt acts *in vitro* as a co‐factor independent methionine synthase

Bmt seems to be involved in shortening the lag phase (Fig. 2A); however, it is still unknown if the enzyme functions together with the other MetH‐like split methionine synthase components (Fig. 1B), or if it can act as an individual enzyme. As outlined in the introduction [16, 17, 31, 32, 33, 34, 35, 37], previous reports support the possibility of direct DMSP demethylation coupled to methionine synthesis by Bmt in *P. inhibens*. Such a direct route would bypass the need for the DMSP demethylase DmdA and the co‐factors THF or cobalamin (Fig. 1B), representing an alternative mechanism for DMSP demethylation to alleviate methionine synthesis in marine bacteria.

**Fig. 3:**
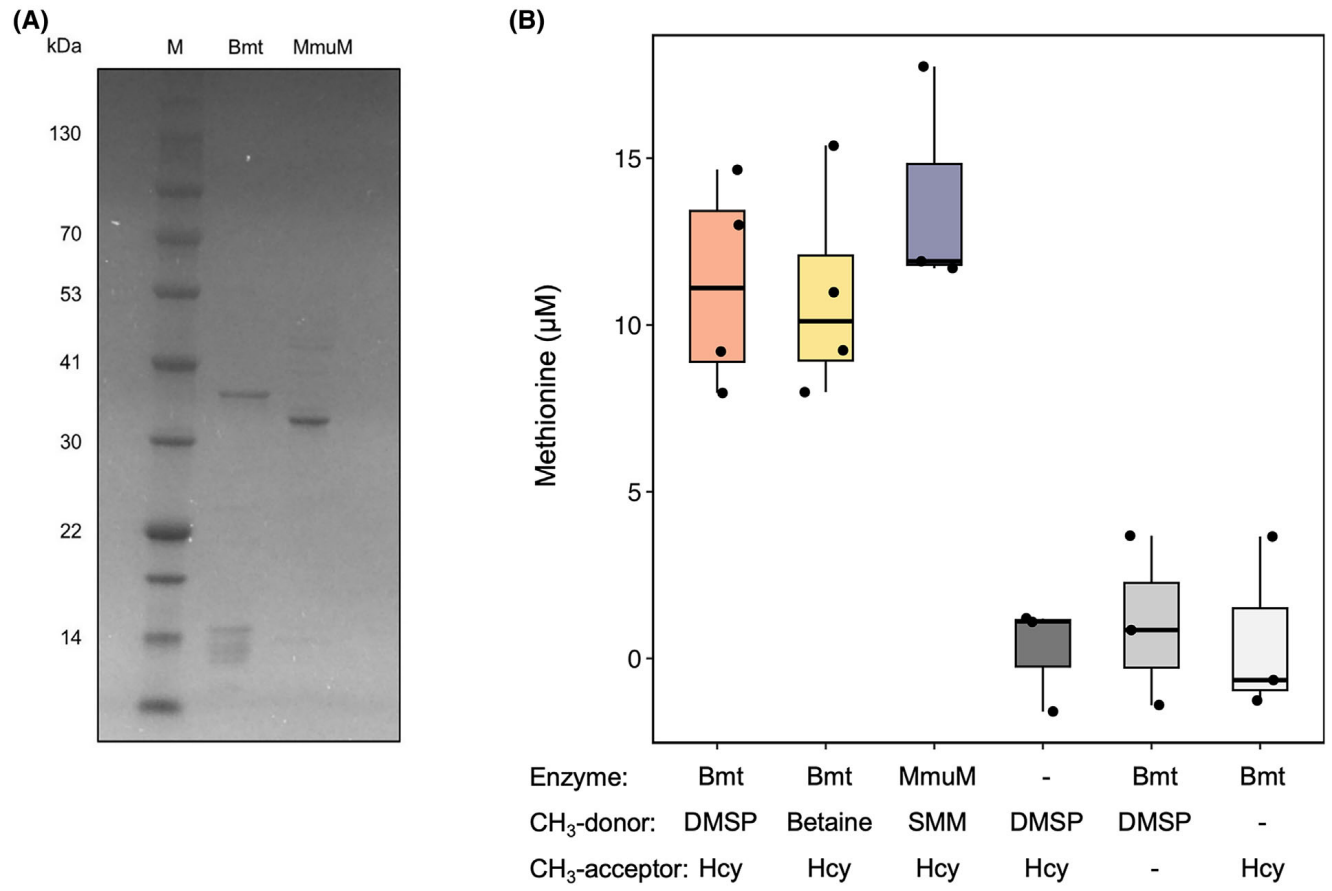
The *Phaeobacter inhibens* Bmt utilizes DMSP and betaine as methyl group donors for coupled methionine synthesis. (A) Purification by affinity chromatography of recombinant methionine synthases. The recombinant *P. inhibens* Bmt (36.06 kDa) and *E. coli* MmuM (33.4 kDa) were separated on a 4–20% Bis‐Tris SDS/PAGE gel using 3 μg protein per lane. Proteins were stained with Coomassie. M—marker. Representative image from at least three biological replicates. (B) The formation of methionine was quantified using *in vitro* reactions containing purified Bmt enzyme together with the methyl group acceptor homocysteine (Hcy) and the methyl group donors DMSP or betaine. The purified methionine synthase MmuM of *E. coli* was used as a positive control, which is similar to Bmt and utilizes *S*‐methylmethionine (SMM) as methyl group donor. Results were compared to negative control reactions in which single components were omitted (either Bmt, Hcy or DMSP). *CH*_*3*_ *‐donor*, methyl group donor; *CH*_*3*_ *‐acceptor*, methyl group acceptor. Box‐plots show results from at least three independent experiments; black dots indicate individual measurements. Box‐plot elements: center line – median; box limits – upper and lower quartiles; whiskers – min and max values.

To investigate whether the *P. inhibens* Bmt enzyme can directly transfer methyl groups from DMSP to homocysteine, we performed an *in vitro* assay. Bmt was heterologously expressed and purified by affinity chromatography (Fig. 3A). The Bmt enzyme was then incubated with homocysteine as a methyl group acceptor and either DMSP or betaine as potential methyl group donors (see Materials and methods for detailed reaction conditions). As a positive control, we included purified *E. coli* MmuM, using SMM as the methyl group donor [17]. Methionine production was quantified using a commercial fluorescence‐based kit. Our results show that Bmt can transfer a methyl group directly from both DMSP and betaine to homocysteine, resulting in methionine production *in vitro* (Fig. 3B), a finding of potential significance considering the limited understanding of betaine demethylases in *P. inhibens*. Thus, *in vitro*, Bmt can function as both a DMSP—homocysteine *S*‐methyltransferase and a betaine—homocysteine *N*‐methyltransferase. While Bmt was previously reported as part of a MetH‐like split methionine synthase *in vivo* [27], our results suggest that the individual Bmt enzyme has the potential to demethylate DMSP and betaine in marine bacteria.

It is important to note, however, that *in vivo*, Bmt does not appear to compensate for the absence of the key demethylase DmdA in the demethylation of DMSP. This conclusion can be drawn from our previous results with a *ΔdmdA* mutant [4]. In this mutant, the DMSP‐dependent lag phase shortening was abolished despite the presence of a *bmt* gene. Taken together, our data demonstrate the potential of the *P. inhibens* Bmt to act *in vitro* as a stand‐alone methionine synthase, using different methylated compounds as methyl group donors. These findings underscore the potential flexibility of the enzymatic machinery responsible for harvesting and assimilating methyl groups from diverse methylated donors.

### Bmt is unable to compensate for the absence of DmdA in shortening the lag phase under standard cultivation conditions

**Fig. 4:**
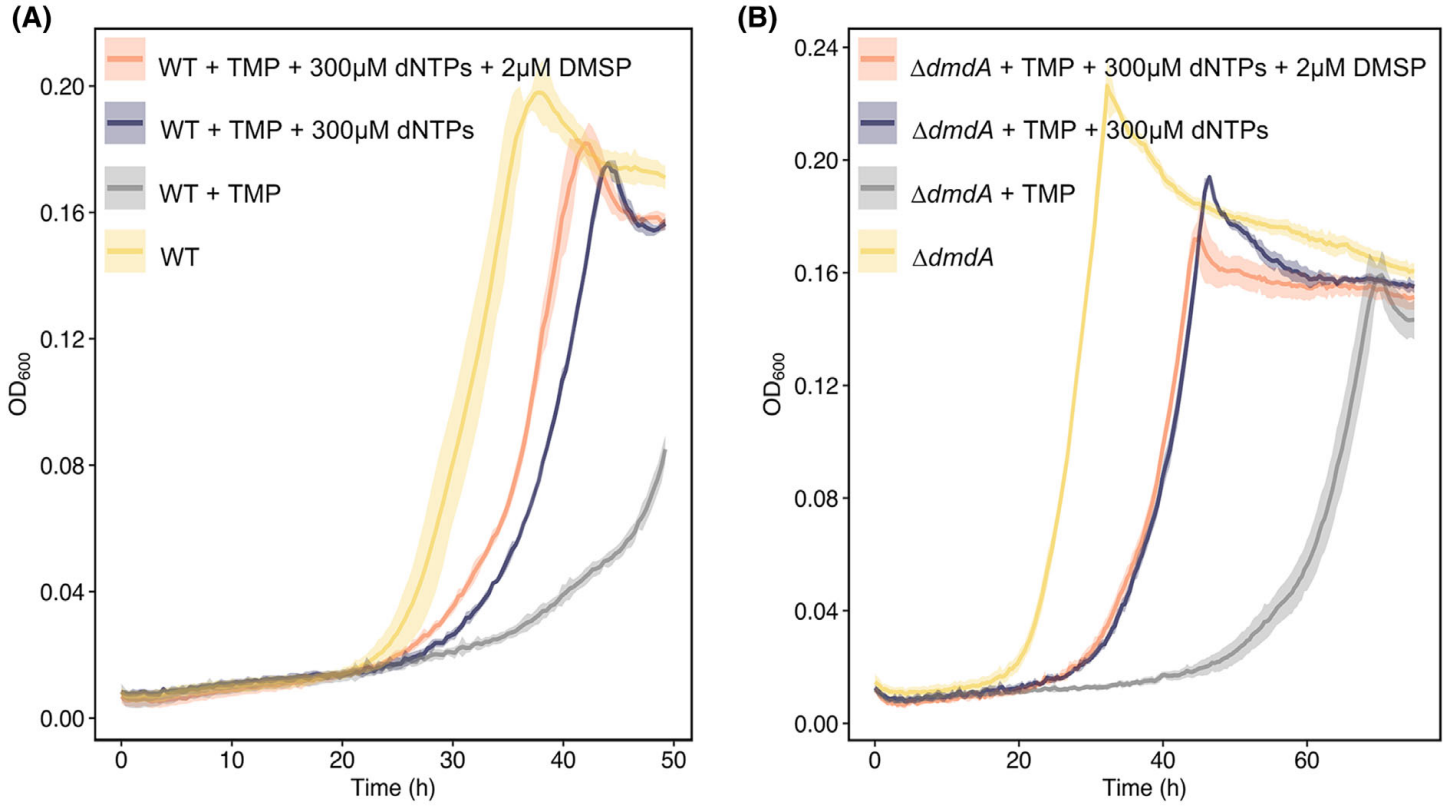
*dmdA* is required for lag phase shortening under standard cultivation conditions. (A) WT and (B) *ΔdmdA* mutant were grown with 50 μg·mL^−1^ trimethoprim (TMP). THF is required for nucleotide synthesis and therefore TMP‐treated cells growth is affected (gray) in contrast with the control (yellow). Bacterial growth was rescued by the external addition of nucleotides (300 μm of dNTPs, purple and orange). WT cultures treated with both TMP and dNTPs exhibited stimulated growth upon supplementation with 2 μm DMSP (orange) compared to control cultures without DMSP (purple). While *ΔdmdA* mutant did not exhibit lag phase shortening. dNTPs, deoxynucleotide triphosphates; *THF*, tetrahydrofolate; *WT*, wild‐type; Δ*dmdA*, dimethylsulfoniopropionate‐dependent demethylase mutant. Lines represent the average growth curve based on three biological replicates and shaded areas indicate the standard deviation (SD).

To further investigate whether Bmt can act *in vivo* as a DMSP—homocysteine *S*‐methyltransferase, potentially leading to methionine synthesis and lag phase shortening, our objective was to inhibit THF‐dependent one‐carbon metabolism in the cell. Such inhibition would disrupt THF‐dependent DMSP demethylation by DmdA, as well as THF‐dependent methionine synthesis by the MetH‐like split methionine synthase. Importantly, inhibition of THF synthesis would not affect the DMSP—homocysteine *S*‐methyltransferase activity of the stand‐alone Bmt enzyme, which can function in a THF‐independent manner (Fig. 3B). Inhibition of THF synthesis was achieved by adding trimethoprim (TMP) to bacterial cultures [44]. TMP, a structural analog of dihydrofolate, inhibits dihydrofolate reductase, an enzyme essential for converting dihydrofolate to the co‐factor tetrahydrofolate [44, 45]. Since TMP also inhibits THF‐dependent nucleotide synthesis, cultures were supplemented with dNTPs to rescue bacterial growth (Fig. 4). Although bacterial growth was significantly disturbed by TMP, bacteria exhibited shorter lag phases in response to supplementation with 2 μm DMSP (Fig. 4A). However, since we observed residual growth in bacteria treated with TMP while not supplemented with nucleotides (gray line), it is possible that the lag shortening may be due to trace amounts of THF. To rule out residual DmdA activity, we repeated the experiments using the *ΔdmdA* mutant strain. Under standard cultivation conditions, mutant *ΔdmdA* bacteria do not exhibit DMSP‐induced lag phase shortening, because DMSP‐derived methyl groups are no longer assimilated via the methionine cycle [4]. Upon addition of TMP to cultures of the *ΔdmdA* mutant strain, no lag phase shortening was detected in response to DMSP (Fig. 4B). Therefore, we conclude that under standard cultivation conditions, DmdA is essential for lag phase shortening and other enzymes, including Bmt, are unable to compensate for its activity.

### Lag phase shortening under stress conditions

**Fig. 5:**
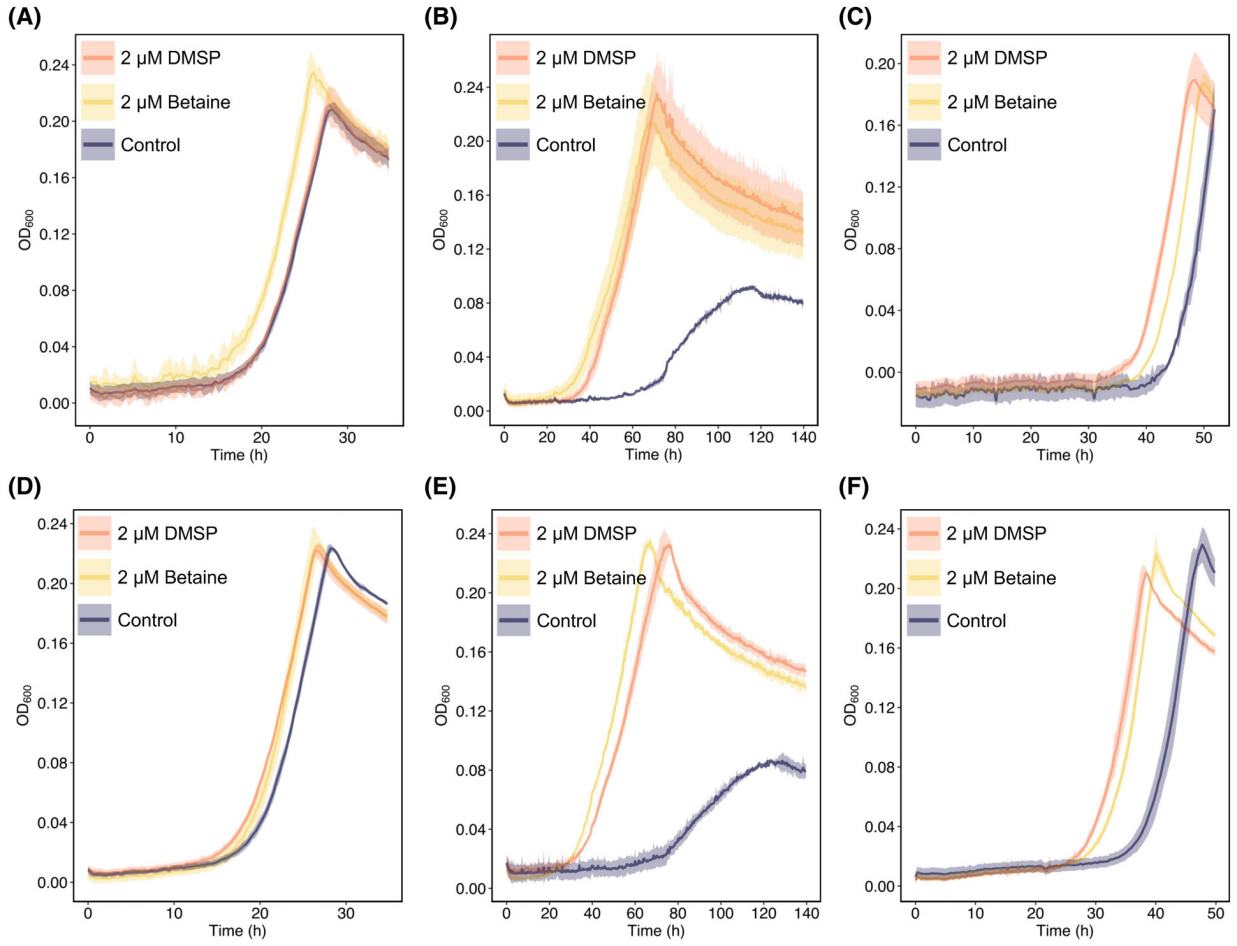
Lag phase shortening is triggered in the ∆*dmdA* mutant and WT bacteria under high salt and oxidative stress conditions. (A) Under standard cultivation conditions in minimal media, the *∆dmdA* mutant responds to betaine supplementation (2 μm, yellow), but not to DMSP (2 μm, orange), similar to the control (purple). However, under (B) high salinity conditions (0.45 m NaCl), and (C) oxidative stress (100 mm H_2_O_2_), DMSP does trigger lag phase shortening in the *∆dmdA* mutant (orange). Lines represent the average growth curve based on three biological replicates and shaded areas indicate the standard deviation (SD). For statistical significance see Table S6. (D) WT bacteria growing under regular cultivation conditions in minimal media (purple) respond to 2 μm DMSP (orange) or betaine supplementation (yellow). This response is enhanced under (E) high salinity (0.45 m NaCl) and (F) oxidative stress (100 μm H_2_O_2_) conditions. Lines represent the average growth curve based on three biological replicates, and shaded areas indicate the standard deviation (SD). For statistical significance see Tables S6 and S7. *H*_*2*_ *O*_*2*_, hydrogen peroxide; *NaCl*, sodium chloride; *WT*, wild‐type; Δ*dmdA*, dimethylsulfoniopropionate‐dependent demethylase mutant.

Our transcriptomic analyses, *in vitro* results, and cultivation experiments with *Δbmt* mutants (Figs 1, 2, 3) suggest that Bmt has the ability to couple DMSP demethylation with methionine synthesis, potentially shortening the bacterial lag phase. However, under standard cultivation conditions, the absence of the primary DMSP demethylase DmdA resulted in no observed lag phase shortening [4]. This suggests that while Bmt may have the potential to contribute to lag phase shortening, the enzyme was not active under these conditions. In the ocean, bacteria frequently experience various environmental stresses such as high salinity and oxidative stress (e.g., H_2_O_2_) [46]. These stress conditions are known to affect methionine synthase activity [16, 38, 47, 48]. Therefore, we tested the *ΔdmdA* mutant under high salinity and oxidative stress conditions to explore whether enzymes other than DmdA, such as Bmt, can support lag phase shortening under these environmental scenarios. To this end, cultures of the *ΔdmdA* mutant were exposed to high osmolarity (0.45 m NaCl) or oxidative stress (100 μm H_2_O_2_). These treatments stress bacteria, as evident by markedly longer lag phases (Fig. 5). The cultures were supplemented with DMSP or betaine (2 μm) or were untreated. Under standard non‐stress conditions, the *ΔdmdA* mutant did not exhibit shorter lag times in response to DMSP, as previously reported [4] (Fig. 5A and Table S6). However, under high osmolarity and oxidative stress conditions, DMSP did trigger lag phase shortening in *ΔdmdA* mutant cultures (Fig. 5B, C, Tables S6 and S8). Additionally, the lag phase shortening induced by DMSP or betaine in WT bacteria was more pronounced under stress compared to standard no‐stress conditions (see Fig. 5D–F and Table S7). These findings suggest that an additional mechanism, distinct from DmdA, contributes to lag phase shortening under stress conditions. This mechanism appears to facilitate lag phase reduction in the *ΔdmdA* strain under stress and likely also influences lag phase dynamics in WT bacteria, which demonstrate a significantly greater reduction in lag time compared to standard cultivation conditions. This observation supports the hypothesis of redundancy or complementarity between DmdA and other enzymes involved in methionine synthesis across varying environmental conditions.

**Fig. 6:**
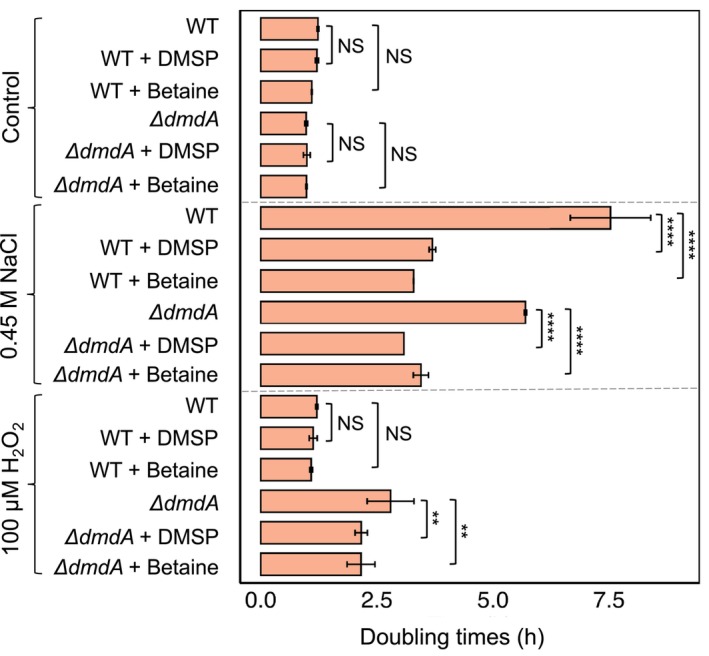
The impact of DMSP and betaine on doubling times of WT and *ΔdmdA Phaeobacter inhibens* under stress conditions. Bacteria were cultivated under salt stress (0.45 m NaCl), oxidative stress (100 mm H_2_O_2_), or standard conditions. Cultures were supplemented with 2 μm DMSP, betaine, or untreated as control, and doubling times were monitored. *H*_*2*_ *O*_*2*_, hydrogen peroxide; *NaCl*, sodium chloride; *WT*, wild‐type; Δ*dmdA*, dimethylsulfoniopropionate‐dependent demethylase mutant. Error bars indicate standard deviations of the mean for three biological replicates. Statistical differences between control and supplemented cultures were assessed with a two‐way ANOVA test followed by a Tukey's test. NS, not significant, **P ≤ 0.004, ****P < 0.0001.

Furthermore, the differential responses to DMSP versus betaine suggest the involvement of distinct cellular mechanisms (Table S8). Under salt stress, the addition of betaine resulted in a more pronounced lag phase shortening in WT bacteria compared to the *ΔdmdA* mutant strain. In contrast, under salt stress, DMSP elicited a similar reduction in lag time in both WT and *ΔdmdA* bacteria. Under oxidative stress, both DMSP and betaine induced a more significant lag phase reduction in WT bacteria compared to the *ΔdmdA* strain. These data indicate a complex interplay between environmental conditions, the type of methylated compound (DMSP versus betaine), and the cellular responses activated in bacteria. Notably, under high salinity conditions, both WT and *ΔdmdA* mutants exhibited prolonged doubling times (i.e., reduced growth rates), with the *ΔdmdA* mutant also showing longer doubling times under oxidative stress (Fig. 6). Under these conditions, the addition of DMSP or betaine had two marked effects: it shortened the lag phase and improved the growth rate by expediting the doubling time. This suggests that under stress conditions, DMSP and betaine may function both as osmoprotectants and as molecules that contribute methyl groups to shorten the lag phase. These observations underscore the bacterial ability to utilize DMSP and betaine in various capacities depending on environmental conditions, as reported previously [16, 49].

**Fig. 7:**
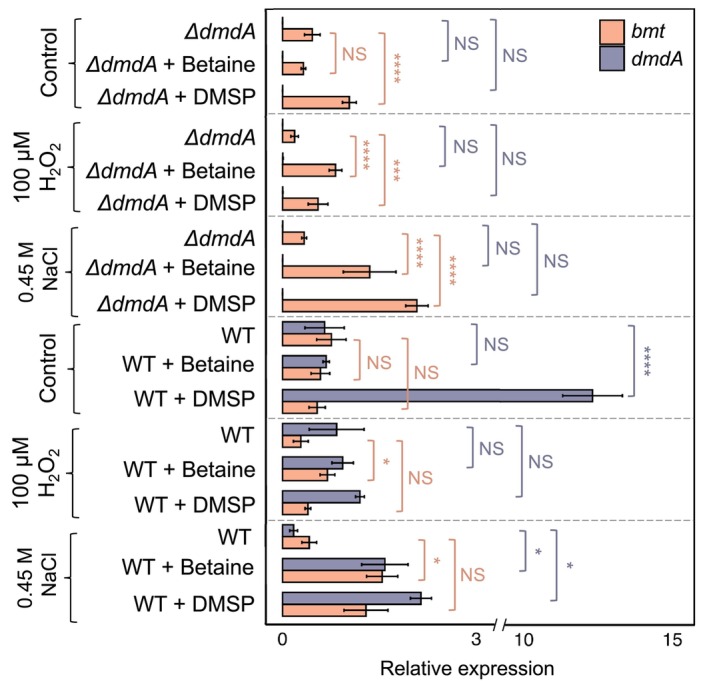
Expression of the *bmt* gene is increased under salt and oxidative stress conditions. Relative gene expression of *bmt* (orange) and *dmdA* (purple) was measured during the lag phase (see Materials and Methods), after 40 min of freshly initiated bacterial cultures under salt stress (0.45 m NaCl), oxidative stress (100 mm H_2_O_2_), or standard conditions (control). Cultures were supplemented with 50 μm DMSP, betaine, or untreated. Bars represent relative expression of *bmt* and *dmdA* normalized to the expression of the housekeeping gene *gyrA*. Error bars indicate standard deviations of the mean for three biological replicates. *dmdA*, dimethylsulfoniopropionate‐dependent demethylase; *H*_*2*_ *O*_*2*_, hydrogen peroxide; *NaCl*, sodium chloride; *WT*, wild‐type; Δ*dmdA*, dimethylsulfoniopropionate‐dependent demethylase mutant. Statistical differences between control and supplemented cultures were assessed with a two‐way ANOVA test followed by a Dunnett's test. NS, not significant, *P ≤ 0.05, ***P = 0.0002, ****P < 0.0001.

To further substantiate the potential involvement of Bmt in lag phase shortening under stress conditions, we profiled the *bmt* expression levels in WT and Δ*dmdA* bacteria during the lag phase. In our previous study, we established the conditions to analyze gene expression during the lag phase in *P. inhibens* [4]. Accordingly, WT and Δ*dmdA* bacterial cultures, grown under salt stress (0.45 m NaCl), oxidative stress (100 mm H_2_O_2_), or standard conditions (control), were supplemented with DMSP or betaine (50 μm), or left untreated. Samples were harvested after 40 min of incubation, and the expression of *bmt* and *dmdA* was analyzed by qRT‐PCR (Real‐Time Quantitative Reverse Transcription PCR). Our data indicate that, under standard (non‐stress) conditions, *dmdA* expression in WT bacteria was upregulated upon DMSP supplementation, as previously reported [4], while *bmt* expression remained unchanged in the presence of both methylated compounds (Fig. 7). Similarly, unchanged expression was observed upon adding betaine to the *ΔdmdA* mutant under standard conditions. Addition of DMSP to the *ΔdmdA* mutant did induce a roughly 2‐fold increase in *bmt* expression under standard conditions, although no lag shortening was observed under these conditions (Fig. 5A). It is important to note that directly comparing the results of gene expression analyses and growth experiments is challenging due to differences in experimental setups (e.g., cell numbers and compound concentrations; see Materials and Methods). In contrast, under oxidative stress conditions, *bmt* expression was induced by betaine in both *ΔdmdA* and WT bacteria, while DMSP triggered *bmt* expression only in the mutant (Fig. 7). Under salt stress, *bmt* was induced by both DMSP and betaine in the *ΔdmdA* mutant, while in WT bacteria, *bmt* was significantly induced by betaine. Under these stress conditions, we also observed the upregulation of *dmdA* in response to both methylated compounds (Fig. 7). These results indicate that *bmt* expression is enhanced during the lag phase under stress conditions in response to methylated compounds, suggesting the potential contribution of Bmt to lag phase shortening under these conditions. Additionally, our data show that *bmt* can be co‐expressed with *dmdA* under stress, suggesting potential complementarity between DmdA and Bmt.

In conclusion, our data indicate that under standard cultivation conditions, DmdA serves as the primary demethylase, transferring a methyl group from DMSP into the methionine cycle, thereby promoting lag phase shortening. However, under osmotic and oxidative stress, additional mechanisms, such as Bmt, may contribute to the utilization of methylated compounds for lag phase reduction.

## Discussion

### The importance and versatility of methyl groups metabolism

Methyl group metabolism, also termed C1 metabolism, plays a central role in cellular physiology, and its disruption is associated with bacterial growth perturbations [9] and various human pathologies [50]. The pathway provides C1 groups to support the biosynthesis of essential molecules such as purine and pyrimidine nucleotides, the amino acids methionine and histidine, as well as polyamines [9, 51]. Methyl groups also serve important regulatory functions and are required to methylate DNA, RNA, and proteins [52, 53, 54]. It was previously shown that the depletion of building blocks results in longer lag phases [3], and that the synthesis of building blocks is upregulated during the lag phase of bacteria [55, 56, 57, 58]. Moreover, impairments in methylation processes can affect critical cellular functions, such as progression through the cell cycle [59], circadian regulation [60] and chemotaxis proteins [61, 62]. Importantly, from a metabolic perspective, generating methyl groups is energetically costly. The synthesis begins with the formation of a methylene group, typically via serine hydroxymethyltransferase and/or the glycine cleavage system [51, 63]. These intermediates are then converted to a methyl groups using NAD(P)H as an electron donor [64]. This, and the requirement for additional co‐factors, position methionine—the only *S*‐methylated amino acid—as the most expensive amino acid to produce [65]. It was shown that *Roseobacter* bacteria cultured on glucose supplemented with DMSP build the majority of their methionine from the methyl groups of exogenous DMSP [66]. Here, we show that Bmt may be directly transferring methyl groups from DMSP to homocysteine for the synthesis of methionine *in vitro* (Fig. 3B). These observations add versatility to the described routes of methionine synthesis [27, 66]. Furthermore, under stress conditions, DMSP and betaine could serve a dual functions, not only acting as an osmoprotectant but also serving as methyl group donors, thereby enhancing the fitness of *P. inhibens*. However, the pathways involved in betaine catabolism in *P. inhibens* remain unclear [28], and further investigation is needed to determine whether such pathways exist and, if so, how they operate at the species level.

### Lag phase‐related regulation of the methionine cycle

The methionine cycle is central in the regulation of the bacterial lag phase. In *P. inhibens*, external supplementation with methionine prolongs the lag phase, rather than shortening it as observed with other methylated compounds [4]. This effect can be either attributed to methionine directly or to the accumulation of intermediates in the methionine cycle, such as S‐adenosylmethionine (SAM), homocysteine, and its precursor, *S*‐adenosylhomocysteine (SAH) [67, 68]. Because this cycle is tightly feedback‐regulated, overproduction of methionine is not sustained in natural bacterial populations [67]. Disruptions in methionine metabolism are also linked to metabolic disorders in higher organisms [69, 70]. In *E. coli*, defects in methionine synthesis are associated with longer lag phases [71], and both methionine and homocysteine have been shown to inhibit growth under certain conditions [38, 72]. Additionally, methionine accumulation can repress the transcription of biosynthetic genes within the cycle [72]. This regulation is further exemplified by MetA, a homoserine O‐succinyltransferase involved in the early biosynthesis of homocysteine, which is subject to negative feedback inhibition by methionine and SAM [39, 73]. Similarly, in *P. inhibens*, methionine supplementation leads to extended lag phases [4].

In line with tight feedback regulation of the methionine cycle, our data show that the methionine synthase gene *bmt* was downregulated in bacteria exposed to DMSP during the lag phase (Fig. 1). A similar pattern was observed for a *bmt* homolog gene in a related *Roseobacter* bacterium when exposed to DMSP (accession: B5M07_08905 [41]). In conclusion, we found that the methionine cycle plays a role in regulating the duration of the lag phase. Our data suggest that an elevated influx of methyl groups into this cycle can be counterbalanced by the downregulation of methionine synthase gene transcription. Results of our experiments with the *Δbmt* mutant (Fig. 2A,B) along with the *bmt* expression profiles (Fig. 7) support the involvement of the Bmt methionine synthase in lag phase shortening induced by methylated compounds.

### Bmt is advantageous under stress conditions

The possible importance of Bmt under conditions of osmotic pressure was previously discussed [16]. It was observed that a mutant strain of the soil bacterium *Sinorhizobium meliloti* lacking the *metH* gene was unable to produce methionine, making it an auxotroph. However, when supplemented with 1 mm betaine, the mutant strain restored its growth, though at slower rates compared to methionine supplementation. This suggested an alternative pathway for methionine synthesis using betaine as a methyl group donor. Further investigation revealed that this pathway is catalyzed by a betaine–homocysteine *N*‐methyltransferase (Bmt), an enzyme previously characterized in humans, rats, and bacteria (BHMT) [16, 74, 75]. Interestingly, under high osmolarity conditions, such as exposure to 0.5 m NaCl, the addition of betaine led to faster growth of the *metH* mutant compared to methionine supplementation alone [16]. While betaine accumulation can be advantageous due to its osmoprotectant properties, these observations suggest that under hyper‐osmotic conditions, Bmt can serve as an efficient demethylase of betaine as well as a methionine synthase, as has been reported for *S. meliloti* [16].

In addition, under high salinity conditions, the addition of DMSP was reported to contribute to the fitness of *Vibrio* bacteria [49]. While there are no reported DMSP demethylases for *Vibrio* species, our previous observations demonstrated that *Vibrio* bacteria encode a *bmt* gene, respond to methylated compounds, and can shorten their lag phase [4]. These data further support a possible link between methionine synthesis using DMSP as a methyl donor and salt stress conditions.

On the other hand, oxidative stress induces methionine auxotrophy in *E. coli* growing in minimal media, caused by the inactivation of the MetE enzyme and resulting in methionine limitation [47]. Additionally, *E. coli* cultures containing methionine resume growth faster after being exposed to oxidative stress conditions [47]. Our data demonstrate that under stress conditions, including high salt and oxidative stress, the *ΔdmdA* mutant is capable of expediting the lag phase possibly by utilizing DMSP as a methyl group donor.

The adaptability of the methionine cycle under stress conditions could provide an advantage for bacteria that constantly face changes in salinity and oxidative stress within the marine environment, especially as these environmental challenges are intensified by anthropogenic activities [46].

### Methionine synthesis in an environmental context

Methionine synthesis is primarily mediated by the cobalamin‐dependent methionine synthase; however, its activity can be compromised by environmental factors like cobalamin deficiency or exposure to nitrous oxide [76, 77]. While some organisms, such as *P. inhibens*, seem to constitutively express genes related to B_12_ biosynthesis [78], B_12_ availability in the ocean is influenced by enzymatic demands and interactions with other cobalamin‐dependent organisms [79, 80]. Despite this knowledge, the specific environmental influences on bacterial methionine synthesis and the corresponding bacterial adaptation strategies remain largely unexplored. For instance, strains of *Vibrio* and *Pseudoalteromonas* exhibit varying capacities to shorten the lag phase in response to DMSP or betaine. A search for the *bmt* gene within the genomes of these bacteria revealed no correlation between their lag shortening abilities and the presence or absence of the *bmt* gene in their reference genomes [4]. This suggests that factors beyond the presence of the *bmt* gene contribute to lag phase shortening. Specifically, bacteria with the *bmt* gene that did not show lag reduction under standard growth conditions may respond differently under stress conditions, as observed in the current study. Additionally, while the metabolic pathway for methionine biosynthesis has been extensively characterized in *E. coli*, many bacterial species diverge from this canonical pathway [8]. Consequently, the full repertoire of genes involved in lag shortening across various marine bacteria remains largely unknown.

Additionally, microbial communities residing on algal cell surfaces, exemplified by the model marine bacterium *P. inhibens* in this study, produce various compounds. This includes bacterial‐produced methionine, which can significantly influence both the fitness of the algal host and the dynamics within the microbial community [77, 80, 81, 82, 83, 84]. Thus, an intricate metabolic interplay exists between microalgae and their associated bacteria, involving the utilization of specific algal methylated compounds and complex algal exudates. Laboratory experiments have indeed demonstrated lag phase shortening in response to specific methylated compounds, as well as to algal supernatants containing the full repertoire of algal exudates [4, 5].

Previously, we have reported that various marine bacteria exhibit different lag phase shortening responses to DMSP and betaine [74]. Several of the tested bacteria, such as *P. inhibens* and *Sulfitobacter pontiacus*, co‐occur in algal microbiomes [21]. Therefore, the observed differences in response to algal metabolites may contribute to shaping the composition of the bacterial population associated with the algal host. In addition, *P. inhibens* has been shown to significantly influence the development of algal‐associated bacterial communities [23]. The ability of *P. inhibens* to grow earlier in response to DMSP or betaine may provide an initial competitive advantage in the process of colonizing the algal host.

To conclude, in the complex web of microbial interactions, methionine synthesis emerges as a crucial metabolic process involved in shaping these relationships.

## Materials and methods

### Media and strains

The bacterial strain *Phaeobacter inhibens* DSM 17395 was purchased from the German Collection of Microorganism and Cell Cultures (DSMZ, Braunschweig, Germany). It was cultivated in ½ YTSS medium (yeast extract, 2 g·L^−1^; trypton, 1.25 g·L^−1^; sea salts; 20 g·L^−1^), or ASW medium based on the protocol of Goyet and Poisson [85] and contained mineral salts (NaCl, 409.41 mm; Na_2_SO_4_, 28.22 mm; KCl, 9.08 mm; KBr, 0.82 mm; NaF, 0.07 mm; Na_2_CO_3_, 0.20 mm; NaHCO_3_, 2 mm; MgCl·6H_2_O, 50.66 mm; CaCl_2_, 10.2 mm, SrCl_2_·6H_2_O, 0.09 mm), L1 trace elements (Na_2_EDTA·2H_2_O, 4.36 mg·L^−1^; FeCl_3_·6H_2_O, 3.15 mg·L^−1^; MnCl_2_·4H_2_O, 178.1 μg·L^−1^; ZnSO_4_·7H_2_O, 23 μg·L^−1^; CoCl_2_·6H_2_O, 11.9 μg·L^−1^; CuSO_4_·5H_2_O, 2.5 μg·L^−1^; Na_2_MoO_4_·2H_2_O, 19.9 μg·L^−1^; H_2_SeO_3_, 1.29 μg·L^−1^; NiSO_4_·6H_2_O, 2.63 μg·L^−1^; Na_3_VO_4_, 1.84 μg·L^−1^; K_2_CrO_4_, 1.94 μg·L^−1^), L1 nutrients (NaNO_3_, 882 μm; NaH_2_PO_4_·2H_2_O, 36.22 μm), 5 mm NH_4_Cl, 33 mm Na_2_SO_4_, and 1 mm Glucose as a carbon source, adjusted to a pH 8.2 with HCl. The same conditions were used for the mutants *Δbmt, Δmes‐like*, *and ΔdmdA*, with the addition of 30 μg·mL^−1^ gentamycin, 150 μg·mL^−1^ kanamycin, and 30 μg·mL^−1^ gentamycin, respectively. Additionally, the mutant *Δbmt* was also supplemented with 200 μm Methionine (Thermo Scientific, Waltham, MA, USA). For high salinity experiments, 0.45 m NaCl (Bio‐Lab Ltd, Jerusalem, Israel) was added to the ASW medium, and for experiments with oxidative stress conditions, 100 μm H_2_O_2_ (Invitrogen, Carlsbad, CA, USA) was added to the ASW medium.

### Bacterial growth curves

Bacteria glycerol stocks were streaked onto ½ YTSS medium agar plates and incubated for 48 h at 30 °C. Then, 10 mL ASW medium was inoculated with a single colony and incubated until reaching stationary phase (48 h at 30 °C with 130 rpm shaking). The pre‐culture was highly diluted (OD_600_ 0.00001) into fresh ASW and supplemented, as indicated, with 2 μm DMSP, 2 μm betaine, 50 μg·mL^−1^ Trimethoprim (TMP, Sigma‐Aldrich, Merck, Darmstadt, Germany), 100 μm dNTPs (Thermo Scientific), 100 μm H_2_O_2_ (Invitrogen), or sterile water as control. Diluted cells were transferred to a 96‐well microtiter plate (150 μL per well) and overlaid with 50 μL hexadecane to prevent evaporation [86]. Bacterial growth was monitored at 30 °C in an Infinite 200 Pro M Plex plate reader (Tecan Group Ltd., Männedorf, Switzerland) with alternating cycles of 5 s shaking and 19:55 min incubation. Absorption measurements were conducted at 600 nm following the shaking step and multiplied by a factor of 3.86 to reflect optical density measurements performed in 1 cm cuvettes. The background absorbance, which is the measured OD_600_ of the culture media without bacterial addition, was subtracted from each individual well. To calculate lag times, we used the formula previously reported by us [4]. To calculate the change in lag times, the mean lag times of supplemented treatment conditions were subtracted from the mean lag time of the control condition.

### Lag phase RNA‐sequencing

The RNA‐sequencing dataset utilized in this study was previously reported by us [4]. Briefly, *P. inhibens* stationary cells were diluted to 0.01 in 30 mL of ASW media and supplemented with 50 μm DMSP or water as control and incubated at 30 °C with 130 rpm shaking. After 15 and 40 min of the inoculation, cells were collected by centrifugation at 4 °C. RNA extracts were obtained with the ISOLATE II RNA Mini Kit (Meridian Bioscience, Cincinnati, OH, USA) and ribosomal rRNA depletion was performed using 100 pmol Pan‐Bacteria probes. The obtained RNA‐sequencing library was sequenced on a NextSeq 500 instrument with a 150 cycles Mid Output Kit (Illumina, San Diego, CA, USA) in paired‐end mode. Quality filtered and trimmed sequencing reads were mapped to *P. inhibens* DSM 17395 genome (accession: GCF_000154765.2). DESeq2 was used for differential gene expression analysis by comparing DMSP‐supplemented samples with control samples 15 and 40 min after inoculation. Raw sequencing data were deposited under the BioProject accession PRJNA977030.

### *Phaeobacter inhibens* Bmt and mes‐like null mutants

The primers and plasmids used for the KO constructs are described in Table 1. DNA manipulation and cloning PCR was performed using Phusion High Fidelity DNA polymerase (Thermo Scientific), according to manufacturer recommended PCR conditions. PCR‐amplified DNA was cleaned with NucleoSpin Gel and PCR Clean‐up kit (MACHEREY‐NAGEL, Düren, Germany). Plasmid DNA was purified using QIAprep Spin Miniprep Kit (QIAGEN, Hilden, Germany).

**Table 1:** Key resources used and generated in this study.

Reagent or resource	Source	Identifier
Bacterial and algal strains		
*Phaeobacter inhibens* DSM 17395; wild type	German Collection of Microorganism and Cell Cultures (DSMZ)	N/A
*Phaeobacter inhibens* DSM 17395; *bmt::aacC1* (Gm^r^) mutant (*Δbmt*)	This paper	ES124
*Phaeobacter inhibens* DSM 17395; *mes‐like::aph* (Km^r^) mutant (*Δmes‐like*)	This paper	ES138
*Escherichia coli* strain BL21(DE3)pLysS	Bacteriology Unit of The Weizmann Institute of Science, Israel	N/A
*Escherichia coli* strain DH5α	Bacteriology Unit of The Weizmann Institute of Science, Israel	N/A
*Escherichia coli* strain K‐12 MG1655	Bacteriology Unit of The Weizmann Institute of Science, Israel	N/A
Chemicals, peptides, and recombinant proteins		
IPTG	ORNAT	Cat#INA‐1758‐1400
Protease Inhibitor Cocktail	Sigma‐Aldrich, Merck	Cat#I3911
Strep‐Tactin™ Sepharose™ Resin	IBA‐Lifesciences	Cat#2–1201‐010
D‐Desthiobiotin	IBA‐Lifesciences	Cat#2–1000‐002
DL‐Dithiothreitol (DTT)	Sigma‐Aldrich, Merck	Cat#DO632; CAS: 3483‐12‐3
Cell Free Amino Acid Mixture – ^13^C,^15^N	Sigma‐Aldrich, Merck	Cat#767964
β‐Mercaptoethanol, 99%	Sigma‐Aldrich, Merck	Cat#M3148 CAS: 60–24‐2
n‐Hexadecane, 99%	Alfa Aeser	Cat#A10322
Critical commercial assays, kits and consumables		
Miracloth	Sigma‐Aldrich, Merck	Cat#475855
Spin‐X^®^ UF concentrators 500 μL, 10 000 MWCO	Corning	Cat#431478
mPAGE 4–20% Bis‐Tris Precast Gel	Sigma‐Aldrich, Merck	Cat#MP42G12
Protein Assay Dye Reagent Concentrate	Bio‐Rad	Cat#5000006
Fluorometric Methionine Assay Kit	Sigma‐Aldrich, Merck	Cat#MAK347
Oligonucleotides		
1. *Δbmt* upstream forward primer with homology to pCR™II‐TOPO™: GTAACGGCCGCCAGTGTGCTGGAATTCGCCCTTGCGCGCTGGCGGGTGCTGC	This paper	N/A
2. *Δbmt* upstream reverse primer with homology to gentamycin cassette: GCATTACAGTTTACGAACCGAACAGGCTTATGTCAAGGGATGTTCCTGTGGGGGTGATATTC	This paper	N/A
3. *Δbmt* downstream forward primer with homology to gentamycin cassette: GGTGGGCTGCCCTTCCTGGTTGGCTTGGTTTCTCATGGACATAGCCCCAAGCCG	This paper	N/A
4. *Δbmt* downstream reverse primer with homology to pCR™II‐TOPO™: CCGCCAGTGTGATGGATATCTGCAGAATTCGCCCTTGGCCCGGTCGTTGATTTCCTAC	This paper	N/A
5. *Δbmt* verification forward primer: CCGGCAGAACGGCGGGCATATCC	This paper	N/A
6. *Δbmt* verification reverse primer: GCTGGGGCGACCTGCGGGATTC	This paper	N/A
7. Gentamycin cassette forward primer: GTTGACATAAGCCTGTTCG	Sperfeld & Narváez‐Barragán *et al*. [4]	N/A
8. Gentamycin cassette reverse primer: GTTAGGTGGCGGTACTTGG	Sperfeld & Narváez‐Barragán *et al*. [4]	N/A
9. Gentamycin cassette verification forward primer: GTGCAAGCAGATTACGGTGACG	Sperfeld & Narváez‐Barragán *et al*. [4]	N/A
10. Gentamycin cassette verification reverse primer: GAGCCTACATGTGCGAATGATGC	Sperfeld & Narváez‐Barragán *et al*. [4]	N/A
11. *Δmes‐like* upstream forward primer with homology to pCR™8/GW/TOPO: GTACAAAAAAGCAGGCTCCGAATTCGCCCTTAGCCTTCATCGACGCCCACAC	This paper	N/A
12. *Δmes‐like* upstream reverse primer with homology to kanamycin cassette: GCATTACAGTTTACGAACCGAACAGGCTTATGTCAAGTGCCGAATCTCCTCAAGTCAG	This paper	N/A
13. *Δmes‐like* downstream forward primer with homology to kanamycin cassette: GCCTTCTATCGCCTTCTTGACGAGTTCTTCTGACACCGCTCCTGCTGCTCCC	This paper	N/A
14. *Δmes‐like* downstream reverse primer with homology to pCR™8/GW/TOPO: CTTTGTACAAGAAAGCTGGGTCGAATTCGCCCTGCCCCAATAGGCGCCAATGC	This paper	N/A
15. *Δmes‐like* verification forward primer: CGGGGTGCGGGTTTGAAACC	This paper	N/A
16. *Δmes‐like* verification reverse primer: GGGCCGATTGATCCGGCAATC	This paper	N/A
17. Kanamycin cassette forward primer: TTGACATAAGCCTGTTCGGTTCG	This paper	N/A
18. Kanamycin cassette reverse primer: TCAGAAGAACTCGTCAAGAAGGCG	This paper	N/A
19. Kanamycin cassette verification forward primer: GAGCACGTACTCGGATGGAA	This paper	N/A
20. Kanamycin cassette verification reverse primer: TTCCATCCGAGTACGTGCTC	This paper	N/A
21. Recombinant *mmuM* forward primer with homology to pET29b: CTTCTCAAATTGAGGATGACTCCATGCACTGCTTCGCGCTTTTAACGCGGC	This paper	N/A
22. Recombinant *mmuM* reverse primer with homology to pET29b: GTTTAACTTTAAGAAGGAGATATACATATGTCGCAGAATAATCCGTTACGCGCTC	This paper	N/A
23. pET29b forward primer with homology to *mmuM*: CAAGAAGAGCGCGTAACGGATTATTCTGCGACATATGTATATCTCCTTCTTAAAGTTAAAC	This paper	N/A
24. pET29b reverse primer with homology to *mmuM*: GCGGATATCGCCGCGTTAAAAGCGCGAAGCAGTGCATGGAGTCATCCTC	This paper	N/A
25. *bmt* forward primer for qRT‐PCR: TATCCACTATGACGGCACGC	This paper	N/A
26. *bmt* reverse primer for qRT‐PCR: GAAGGGACCAAGCACCTCAA	This paper	N/A
27. *dmdA* forward primer for qRT‐PCR: CGTGCAAGTTTGGGATGTGG	This paper	N/A
28. *dmdA* reverse primer for qRT‐PCR: CCAGCGGGTGTCACTATGTT	This paper	N/A
29. *gyrA* forward primer for qRT‐PCR: GCCGATTCCTGACCTCCTTC	Lipsman *et al*. [5]	N/A
30. *gyrA* forward primer for qRT‐PCR: TCAGCTTATGTCGGGCTTCG	Lipsman *et al*. [5]	N/A
Recombinant DNA		
pDN4: *Δbmt* plasmid (Upstream *bmt*‐Gm‐Downstream *bmt‐*TOPOII)	This paper	N/A
pDN11: *Δmes‐like* plasmid (Upstream *mes‐like*‐Km‐Downstream *mes‐like*‐TOPO8)	This paper	N/A
pDN3: *bmt* expression vector (StrepTagII‐*bmt*‐pET29b)	This paper	Twist Biosciences
pDN10: *mmuM* expression vector (*mmuM*‐StrepTagII‐pET29b)	This paper	N/A
pYDR1	Abada *et al*. [24]	N/A
pCR™8/GW/TOPO^®^	Invitrogen	Cat#K250020

For creation of *bmt* null mutant (*Δbmt*) cells (ES124), ~ 1000 bp regions upstream and downstream of the *bmt* gene (accession: PGA1_c13370) were amplified by PCR, using primers 1, 2, 3, and 4, respectively. The gentamycin resistance marker of pBBR1MCS5 was amplified using primers 7 and 8. The PCR‐amplified fragments (upstream region + gentamycin resistance + downstream region) were assembled and cloned into the pCR™II‐TOPO™ vector (Invitrogen, Thermo Fisher Scientific, Waltham, MA, USA) using restriction‐free cloning [87], generating the plasmid pDN4.

For creation of *mes‐like* null mutant (*Δmes‐like*) cells (ES138), ~ 1000 bp regions upstream and downstream of the *mes‐like* gene (accession: PGA1_c27430) were amplified by PCR, using the primers 11, 12, 13, and 14 respectively. The kanamycin resistance marker of pYDR1 was amplified using primers 17 and 18. The PCR‐amplified fragments (upstream region + kanamycin resistance + downstream region) were assembled and cloned into the pCR™8/GW/TOPO^®^ vector (Invitrogen) using restriction‐free cloning [87], generating the plasmid pDN11.

*Phaeobacter inhibens* electrocompetent cells (300 μL) were transformed with 10 μg of the constructed plasmids by a pulse of 2.5 kV (Bio‐Rad, Hercules, CA, USA), and cells were selected on ½ YTSS plates containing 30 μg·mL^−1^ Gentamycin or 150 μg·mL^−1^ Kanamycin. Successful null mutants were verified in single cell clones by PCR (5, 6, 9, 10 for *Δbmt* KO, and 15, 16, 19, 20 for *Δmes‐like*) and sanger sequencing.

### Methionine synthesis with purified Bmt

Details of primers and plasmids used for heterologous expression of Bmt and MmuM (used as a positive control) are provided in Table 1. The protein purification and *in vitro* methionine synthesis were performed as previously described by us [88]. For the production of *P. inhibens* Bmt (accession: PGA1_c13370), the gene was cloned in a pET29b expression vector, incorporating an N‐terminal Strep‐tag II peptide sequence for downstream purification (pDN3). The vector was synthesized by Twist Biosciences (San Francisco, CA, USA). To express *E. coli* MmuM (accession: Q47690), the gene was amplified from *E. coli* K‐12 MG1655 (primers 27–28). The vector was amplified from pET29b (primers 29–30), and both fragments were assembled using circular polymerase extension cloning (CPEC) [89]. This resulted in a vector encoding a C‐terminally tagged MmuM (pDN10). All plasmids were validated by Sanger sequencing. Expression vectors were introduced into *E. coli* BL21 competent cells by electroporation with 100 μg of plasmid DNA, applying a 1.8 kV pulse (MicroPulser, Bio‐Rad Laboratories). Transformants were selected on LB medium agar plates supplemented with 50 μg·mL^−1^ kanamycin.

For Bmt and MmuM protein production, *E. coli* BL21 transformants were cultivated in TYG medium supplemented with 50 μg·mL^−1^ kanamycin at 37 °C until reaching OD_600_ = 0.7 corresponding to mid‐log phase. Protein expression was induced with 0.2 mm IPTG. After 3 h, cells were harvested by centrifugation (3100 ***g***, 15 min, 4 °C). Cell pellets were then resuspended in 20 mL NP buffer (50 mm NaH_2_PO_4_, 300 mm NaCl, pH 8) with 100 μL 10X Protease Inhibitor Cocktail (Sigma‐Aldrich, Merck). Cell suspensions were filtered using Miracloth (Sigma‐Aldrich, Merck) and disrupted by three passes through a French Pressure cell press (15 000 psi). The lysate was centrifuged (3100 ***g***, 15 min, 4 °C) and the supernatant was loaded onto a Strep‐Tactin Sepharose resin column (IBA‐Lifesciences, Göttingen, Germany). The column was washed with 5 mL NP buffer three times, and bound proteins were eluted with 3 mL NPD buffer (50 mm NaH_2_PO_4_, 300 mm NaCl, 2.5 mm Desthibiotin, pH 8). Eluted proteins were concentrated in 20 mm HEPES‐KOH buffer (pH 7.5) using a 10 kDa membrane (Spin‐X^®^ UF 500 μL Centrifugal Concentrator, 10 000 MWCO; Corning, NY, USA). Protein concentration was measured using the Protein Assay Dye Reagent Concentrate (Bio‐Rad Laboratories). The purity was assessed by SDS/PAGE (mPAGE™ 4–20% Bis‐Tris Precast Gel, Merck, Darmstadt, Germany), by loading 3 μg of protein with SDS‐sample buffer, followed by Bio‐Safe Coomassie staining (Bio‐Rad Laboratories).

Methionine synthesis with either DMSP or betaine as methyl group donors was measured *in vitro*, using purified enzyme and a protocol adapted from Ranocha *et al*. [90]. The *in vitro* reactions contained buffer (20 mm HEPES‐KOH, pH 7.5, 2 mm dithiothreitol), a methyl group acceptor (2 mm homocysteine), a methyl group donor (200 μm of either DMSP, betaine or SMM) and purified enzyme (200 μm of either Bmt or MmuM). The *in vitro* reactions (v = 50 μL) were incubated for 2 h at 30 °C. Methionine synthesis was measured using the Methionine Assay Kit (Sigma‐Aldrich, Merck). For measurements, 20 μL of each *in vitro* reaction was mixed with 30 μL Met Assay Buffer and 50 μL Met Assay Reaction Mix. In parallel, 20 μL of the same *in vitro* reactions were mixed with 30 μL Met Assay Buffer and 50 μL Met Assay Background Control Mix. The mixes were incubated for 30 min at 37 °C, followed by fluorescence readings (λ_ex_ = 535 nm/λ_em_ = 587 nm; Infinite 200 Pro M Plex plate reader, Tecan Group Ltd.). The Methionine Assay Kit includes a methionine standard for absolute quantifications. The steps needed to produce purified proteins and to measure enzymatic methionine formation were repeated in three independent experiments.

### *Phaeobacter inhibens* bmt and dmdA gene expression

To analyze the expression changes in *bmt* and *dmdA* genes during the lag phase of *P. inhibens*, the bacterial inoculum (OD_600_) and methyl donor concentrations were adjusted to obtain sufficient biomass for the experiments, while maintaining the expression of C1 metabolism‐related genes, as previously reported [4]. Bacterial pre‐cultures (as described in *Bacterial growth curves*) of WT and *ΔdmdA* mutant were diluted (OD_600_ 0.01) to a final volume of 50 mL of fresh ASW (standard conditions), ASW with 0.45 m NaCl (salt stress) or ASW with 100 mm H_2_O_2_ (oxidative stress), supplemented with 50 μm DMSP, 50 μm betaine, or sterile water as control. Bacterial cultures were incubated at 30 °C with 130 rpm shaking. After 40 min, cells were harvested by centrifugation at 3100 ***g*** for 10 min. Cell pellets were resuspended in RNA lysis buffer (RLT, QIAGEN) with 1% β‐mercaptoethanol and transferred into tubes containing 300 mg of 100 μm acid‐washed Low Binding Silica Beads (SPEX SamplePrep), and disrupted by bead beating at 30 mHz for 3 min. RNA was extracted using the Isolate II RNA Mini Kit (Meridian Bioscience, London, UK). The extracted RNA was subjected to a second DNAse treatment with TURBO DNase kit (Thermo Fisher Scientific) and subsequently purified using RNAClean XP magnetic beads (Beckman Coulter). Total RNA was measured with the Qubit RNA HS Assay (Invitrogen, Thermo Fisher Scientific). Equal concentrations of RNA were used for the cDNA synthesis of all the samples using Superscript IV (ThermoFisher). Quantitative PCR (qPCR) was performed using the SensiFAST SYBR Lo‐ROX Kit (Meridian Bioscience) in a 384‐well plate on a QuantStudio 5 Real‐Time PCR system (Applied Biosystems, Foster City, CA, USA). Each reaction followed a 40‐cycle amplification protocol, optimized according to the manufacturer's recommendations. Primer performance was validated using a serial dilution of pooled cDNA. Only primer pairs with amplification efficiencies of at least 85% were included in the analysis. Relative gene expression was quantified using the Comparative CT (ΔΔ*C*_T_) method, with *gyrA* serving as the internal reference for normalization [5].

## Conflict of interest

The authors have a pending patent application PCT/IL2023/050169. The authors declare no conflict of interest.

## Author contributions

DANB, MS, and ES designed the study and wrote the manuscript. DANB and MS performed and analyzed experiments.

## Peer review

The peer review history for this article is available at https://www.webofscience.com/api/gateway/wos/peer‐review/10.1111/febs.70128.

## Supporting information


**Table S1.** Correlations between the expression of split methionine synthase genes.
**Table S2.** Expression of methionine synthesis‐related genes.
**Table S3.** DMSP does not impact the lag phase duration in the *Δbmt* mutant.
**Table S4.** Lag phase reduction in WT bacteria treated with DMSP and methionine.
**Table S5.** Homology Template Sequences for *P. inhibens* Mes‐like.
**Table S6.** Lag phase reduction in the *ΔdmdA* mutant.
**Table S7.** Lag phase reduction in WT bacteria under standard or stress conditions.
**Table S8.** Lag phase reduction in WT bacteria and *ΔdmdA* mutants under stress conditions.

## Data Availability

This study includes no data deposited in external repositories. The RNA‐sequencing source data utilized in this study was previously deposited under the BioProject accession ID PRJNA77030 [4].
