# Supplementary material for: DmdA‐independent lag phase shortening in Phaeobacter inhibens bacteria under stress conditions
Source: FEBS J. 2025 May 3;292(16):4232–53. doi: 10.1111/febs.70128 (PMC12366255; doi:10.1111/febs.70128)
Supplement: Supplementary file 1 — Table S1. Correlations between the expression of split methionine synthase genes. Table S2. Expression of methionine synthesis‐related genes. Table S3. DMSP does not impact the lag phase duration in the Δbmt mutant. Table S4. Lag phase reduction in WT bacteria treated with DMSP and methionine. Table S5. Homology Template Sequences for P. inhibens Mes‐like. Table S6. Lag phase reduction in the ΔdmdA mutant. Table S7. Lag phase reduction in WT bacteria under standard or stress conditions. Table S8. Lag phase reduction in WT bacteria and ΔdmdA mutants under stress conditions. [file FEBS-292-4232-s001.pdf]

table S1. Correlations between the expression of split methionine synthase genes. Pearson correlations were calculated, with *p*-values adjusted using a false discovery rate of 5%. The underlying dataset was previously described in Sperfeld & Narváez-Barragán *et al.*[1]

**Genes**	**Pearson correlation**	**Adjusted *p*-value**
metH vs *cbp*	-0.13	0.69
metH vs *bmt*	0.90	$5.1*10^{-5}$
bmt vs *cbp*	-0.44	0.15

table S2. Expression of methionine synthesis-related genes. Log2 Fold changes of methionine synthesis related genes during the lag phase of *P. inhibens* bacteria supplemented with 50 µM DMSP compared to control cultures (Fig. 1C). The underlying dataset was previously described in Sperfeld & Narvaez-Barragan *et al.*[1]

**Genes**	**Control vs. DMSP**			
	**15 min post initiation**		**40 min post initiation**	
	**Log2 Fold change**	**Adjusted *p*-value**	**Log2 Fold change**	**Adjusted *p*-value**
cpb (PGA1_c13350)	1.05	0.00	0.50	0.00
(PGA1_c16040)	0.13	0.18	-0.26	0.00
Mes-like (PGA1_c27430)	-1.23	0.00	-0.22	0.20
dmdA (PGA1_262p01830)	3.04	0.00	4.34	0.00
bmt (PGA1_c13370)	-0.78	0.00	-1.57	0.00

table S3. DMSP does not impact the lag phase duration in the *Δbmt* mutant. The duration of the lag phase is unaffected by 2 µM DMSP supplementation in the *Δbmt* mutant, whether grown in the presence or absence of 200 µM Methionine. Values in brackets indicate the standard deviation of the difference between estimated means, using three biological replicates per condition. A t-test with Welch's correction was used to determine differences between treatments.

**Treatment**	**Lag duration (h)**	***p*-value**
Δbmt	30.8 (±1)	0.88
Δbmt + 2 µM DMSP	30.9 (±0.9)	
Δbmt + 200 µM Met	24.2 (±0.9)	0.67
Δbmt + 200 µM Met + 2 µM DMSP	24.6 (±0.6)	

table S4. Lag phase reduction in WT bacteria treated with DMSP and methionine. Cultures of WT *P. inhibens* bacteria were supplemented with 2 µM DMSP and with or without 200 µM methionine (compared with control cultures supplemented with water). Values in brackets indicate the standard deviation of the difference between estimated means, using three biological replicates per condition. A *t*-test with Welch's correction was used to determine differences between treatments.

Treatment	Lag reduction (h)	*P*-value
WT + 2 µM DMSP	3.5 (±1.4)	0.02
WT + 200 µM Methionine + 2 µM DMSP	3 (±1)	0.04

table S5. Homology Template Sequences for *P. inhibens* Mes-like. Templates were searched using BLAST and HHblits against the SWISS-MODEL template library[2].

Template	Seq Identity	Oligo-state	QSQE	Found by	Method	Resolution	Seq Similarity	Coverage	Description
Q161E7.1.A	83.16	monomer	-	AFDB search	AlphaFold v2	NA	0.56	1.00	5-methyltetrahydropteroyltriglutamate--homocysteine methyltransferase, putative
4ztx.1.A	19.82	monomer	-	HHblits	X-ray	2.10Å	0.29	0.87	Cobalamin-Independent Methionine synthase
1t7l.2.A	17.74	monomer	-	HHblits	X-ray	2.00Å	0.30	0.87	5-methyltetrahydropteroyltriglutamate--homocysteine methyltransferase
3rpd.1.A	22.02	monomer	-	HHblits	X-ray	1.50Å	0.31	0.87	Methionine synthase (B12-independent)
1xpg.1.A	17.43	monomer	-	HHblits	X-ray	2.59Å	0.30	0.87	5-methyltetrahydropteroyltriglutamate--homocysteine methyltransferase
1xr2.1.A	18.04	monomer	-	HHblits	X-ray	2.35Å	0.30	0.87	5-methyltetrahydropteroyltriglutamate--homocysteine methyltransferase
4qqu.1.A	17.85	monomer	-	HHblits	X-ray	2.98Å	0.29	0.87	5-methyltetrahydropteroyltriglutamate--homocysteine methyltransferase
3ppc.1.A	17.85	monomer	-	HHblits	X-ray	2.20Å	0.29	0.87	5-methyltetrahydropteroyltriglutamate--homocysteine methyltransferase
3l7r.1.A	19.57	monomer	-	HHblits	X-ray	2.40Å	0.30	0.87	5-methyltetrahydropteroyltriglutamate--homocysteine methyltransferase

3pph.1.A	17.85	monomer	-	HHblits	X-ray	2.80 Å	0.29	0.87	5-methyltetrahydropteroyltriglutamate--homocysteine methyltransferase
1u1h.1.A	18.90	monomer	-	HHblits	X-ray	2.55 Å	0.30	0.87	5-methyltetrahydropteroyltriglutamate--homocysteine methyltransferase
1ypx.1.A	17.90	monomer	-	HHblits	X-ray	2.60 Å	0.29	0.94	putative vitamin-B12 independent methionine synthase family protein
3ppc.2.A	17.85	monomer	-	HHblits	X-ray	2.20 Å	0.29	0.87	5-methyltetrahydropteroyltriglutamate--homocysteine methyltransferase
3ppg.1.A	17.85	monomer	-	HHblits	X-ray	1.98 Å	0.29	0.87	5-methyltetrahydropteroyltriglutamate--homocysteine methyltransferase
2nq5.1.A	19.57	monomer	-	HHblits	X-ray	1.90 Å	0.30	0.87	5-methyltetrahydropteroyltriglutamate--homocysteine methyltransferase
3ppg.1.A	16.31	monomer	-	HHblits	X-ray	1.98 Å	0.27	0.88	5-methyltetrahydropteroyltriglutamate--homocysteine methyltransferase
1u1h.1.A	15.48	monomer	-	HHblits	X-ray	2.55 Å	0.27	0.90	5-methyltetrahydropteroyltriglutamate--homocysteine methyltransferase
2nq5.1.A	14.68	monomer	-	HHblits	X-ray	1.90 Å	0.27	0.87	5-methyltetrahydropteroyltriglutamate--homocysteine methyltransferase
4qqu.1.A	16.31	monomer	-	HHblits	X-ray	2.98 Å	0.27	0.88	5-methyltetrahydropteroyltriglutamate--homocysteine methyltransferase
3ppc.1.A	16.31	monomer	-	HHblits	X-ray	2.20 Å	0.27	0.88	5-methyltetrahydropteroyltriglutamate--homocysteine methyltransferase
3ppc.2.A	16.31	monomer	-	HHblits	X-ray	2.20 Å	0.27	0.88	5-methyltetrahydropteroyltrigluta
1u1u.1.A	15.48	monomer	-	HHblits	X-ray	2.95 Å	0.27	0.90	5-methyltetrahydropteroyltriglutamate--homocysteine methyltransferase
1xr2.1.A	13.84	monomer	-	HHblits	X-ray	2.35 Å	0.27	0.85	5-methyltetrahydropteroyltriglutamate--homocysteine methyltransferase
1t7l.2.A	13.84	monomer	-	HHblits	X-ray	2.00 Å	0.27	0.85	5-methyltetrahydropteroyltriglutamate--homocysteine methyltransferase
1xpg.1.A	13.84	monomer	-	HHblits	X-ray	2.59 Å	0.27	0.85	5-methyltetrahydropteroyltriglutamate--homocysteine methyltransferase
1u1u.1.A	18.90	monomer	-	HHblits	X-ray	2.95 Å	0.30	0.87	5-methyltetrahydropteroyltriglutamate--homocysteine methyltransferase

3rpd.1.A	27.86	monomer	-	BLAST	X-ray	1.50 Å	0.35	0.75	Methionine synthase (B12-independent)
6w2o.1.A	15.85	monomer	-	HHblits	X-ray	1.55 Å	0.27	0.44	Uroporphyrinogen decarboxylase
1r3w.1.A	18.79	monomer	-	HHblits	X-ray	1.70 Å	0.28	0.44	Uroporphyrinogen Decarboxylase
1r3s.1.A	18.79	monomer	-	HHblits	X-ray	1.65 Å	0.28	0.44	Uroporphyrinogen Decarboxylase
1jpi.1.A	18.79	homo-dimer	-	HHblits	X-ray	2.30 Å	0.28	0.44	UROPORPHYRINOGEN DECARBOXYLASE
4wsh.1.A	14.02	homo-dimer	-	HHblits	X-ray	1.95 Å	0.27	0.44	Uroporphyrinogen decarboxylase
1jpk.1.A	18.79	homo-dimer	-	HHblits	X-ray	2.20 Å	0.28	0.44	UROPORPHYRINOGEN DECARBOXYLASE
1j93.1.A	17.28	homo-dimer	-	HHblits	X-ray	2.30 Å	0.28	0.43	UROPORPHYRINOGEN DECARBOXYLASE
3gw3.1.A	18.79	homo-dimer	-	HHblits	X-ray	1.70 Å	0.28	0.44	Uroporphyrinogen decarboxylase
1r3q.1.B	18.90	monomer	-	HHblits	X-ray	1.70 Å	0.28	0.44	Uroporphyrinogen Decarboxylase
3gvv.1.A	18.90	homo-dimer	-	HHblits	X-ray	2.80 Å	0.28	0.44	Uroporphyrinogen decarboxylase
1jph.1.A	18.90	homo-dimer	-	HHblits	X-ray	2.10 Å	0.28	0.44	UROPORPHYRINOGEN DECARBOXYLASE
1r3v.1.A	17.96	monomer	-	HHblits	X-ray	1.90 Å	0.28	0.45	Uroporphyrinogen Decarboxylase
2q6z.1.A	18.79	homo-dimer	-	HHblits	X-ray	2.00 Å	0.28	0.44	Uroporphyrinogen decarboxylase
8d45.1.A	19.05	monomer	-	HHblits	EM	NA	0.29	0.22	Betaine--homocysteine S-methyltransferase 1
4m3p.1.D	19.05	monomer	-	HHblits	X-ray	1.89 Å	0.29	0.22	Betaine--homocysteine S-methyltransferase 1
4m3p.1.A	19.05	monomer	-	HHblits	X-ray	1.89 Å	0.29	0.22	Betaine--homocysteine S-methyltransferase 1
7fev.1.A	13.46	monomer	-	HHblits	X-ray	1.59 Å	0.26	0.28	FMN binding
6bhl.1.B	18.75	monomer	-	HHblits	X-ray	1.40 Å	0.29	0.09	Phosphotriesterase
6bhl.1.A	18.75	monomer	-	HHblits	X-ray	1.40 Å	0.29	0.09	Phosphotriesterase
1ez2.1.B	20.00	monomer	-	HHblits	X-ray	1.90 Å	0.29	0.08	PHOSPHOTRIESTERASE

table S6. Lag phase reduction in the *ΔdmdA* mutant. Lag phase reduction in *ΔdmdA* mutant by 2 µM DMSP or betaine (compared with controls with water) under normal or stress conditions. Values in brackets indicate the standard deviation of the difference between estimated means, using three biological replicates per condition. One-way ANOVA was used to determine differences between groups, followed by Tukey's multiple comparison test, to identify specific group differences between control and supplemented. NS: Not significant.

Treatment	Lag reduction (h)	*p-value*
ΔdmdA + 2 µM DMSP	0.1 (±0.5)	NS
ΔdmdA + 2 µM Betaine	3.2 (±0.2)	0.002
ΔdmdA + 0.45 M NaCl + 2 µM DMSP	27.1 (±1.8)	<0.0001
ΔdmdA + 0.45 M NaCl + 2 µM Betaine	32.4 (±0.4)	<0.0001
ΔdmdA + 100 µM H_2O_2 + 2 µM DMSP	6 (±0.3)	<0.0001
ΔdmdA + 100 µM H_2O_2 + 2 µM Betaine	3.2 (±0.1)	0.002

table S7. Lag phase reduction in WT bacteria under standard or stress conditions. Lag phase shortening in WT bacteria under standard or stress conditions was monitored upon addition of 2 µM DMSP or betaine (compared with controls with water). Values in brackets indicate the standard deviation of the difference between estimated means, using three biological replicates per condition. One-way ANOVA was used to determine differences between groups, followed by Tukey's multiple comparison test, to identify specific group differences between control and supplemented.

Treatment	Lag reduction (h)	*p-value*
WT + 2 µM DMSP	2.7 (±0.4)	0.002
WT + 2 µM Betaine	1.8 (±0.3)	0.01
WT + 0.45 M NaCl + 2 µM DMSP	30.4 (±0.4)	<0.0001
WT + 0.45 M NaCl + 2 µM Betaine	35.1(±0.1)	<0.0001
WT + 100 µM H_2O_2 + 2 µM DMSP	7.8 (±0.1)	0.003
WT + 100 µM H_2O_2 + 2 µM Betaine	5.9 (±0.4)	0.004

table S8. Lag phase reduction in WT bacteria and *ΔdmdA* mutants under stress conditions. Lag phase shortening was induced by 2 μM DMSP or betaine (compared to controls with water) under salt and oxidative stress conditions. Values in brackets indicate the standard deviation of the difference between estimated means, using three biological replicates per condition. ND, non detected. A *t*-test with Welch's correction was used to determine differences between treatments.

Treatment	Lag reduction (h)		WT vs *ΔdmdA* *p*-value	
	DMSP	Betaine	DMSP	Betaine
WT + 0.45 M NaCl	30.4 (±0.4)	35.1(±0.1)	0.1	0.008
ΔdmdA + 0.45 M NaCl	27.1 (±1.8)	32.4 (±0.4)		
WT + 100 μM H_2O_2	7.8 (±0.1)	5.9 (±0.4)	0.009	0.03
ΔdmdA + 100 μM H_2O_2	6 (±0.3)	3.2 (±0.1)		

References for Supplementary Materials

1. Sperfeld, M., Narváez-Barragán, D.A., Malitsky, S., Frydman, V., Yuda, L., Rocha, J., and Segev, E. (2024). Algal methylated compounds shorten the lag phase of *Phaeobacter inhibens* bacteria. Nat Microbiol, 1–16. https://doi.org/10.1038/s41564-024-01742-6.

2. Waterhouse, A., Bertoni, M., Bienert, S., Studer, G., Tauriello, G., Gumienny, R., Heer, F.T., de Beer, T.A.P., Rempfer, C., Bordoli, L., et al. (2018). SWISS-MODEL: homology modelling of protein structures and complexes. Nucleic Acids Research *46*, W296–W303. https://doi.org/10.1093/nar/gky427.
